# Persistent effect at 30-month post intervention of a community-based randomized trial of KM2H^2^ in reducing stroke and heart attack among senior hypertensive patients

**DOI:** 10.1186/s12966-017-0635-3

**Published:** 2018-01-02

**Authors:** Jie Gong, Yunan Xu, Xinguang Chen, Niannian Yang, Fang Li, Yaqiong Yan

**Affiliations:** 1 Wuhan Center for Disease Prevention and Control, 24 Jianghan North Road, Wuhan, 430022 China; 2 Department of Epidemiology, University of Florida, 2004 Mowry Road, Gainesville, Florida, 32610 USA

**Keywords:** Physical activity, Stroke and heart attack, Hypertension, Multicenter trial

## Abstract

**Background:**

The effect of the Keep Moving toward Healthy Heart and Healthy Brain (KM2H2) program at 6-month post intervention has been assessed.  The purpose of this study is to evaluate the KM2H^2^ program at 30-month post intervention.

**Methods:**

A total of 450 senior hypertensive patients from 12 community health centers were randomized by center to either receive KM2H^2^ plus standard care (6 centers, *n* = 232) or standard care only (6 centers, *n* = 218). Data for outcome measures at 30-month post intervention were analyzed. New cases of stroke and heart attack were verified with medical records; levels of physical activity were assessed using self-reported questionnaire. In addition to comparative analysis, adjusted incidence rate and program effects were determined using mixed effects modeling method.

**Results:**

At the 30-month follow-up, the adjusted incidence rate [95% CI] of stroke was 11.81% [5.90, 17.72] for patients in the intervention group and 19.78% [14.07, 25.50] (*p* = 0.03) for the control group. The adjusted incidence rate of heart attack was 3.34% [1.91, 8.58] and 6.68% [1.64, 11.73] for the intervention and control groups (*p* = 0.16), respectively; the proportion and the duration of engaging in regular physical activity were significantly greater for the intervention group than the control group. The reductions in blood pressure between the intervention and the control was not statistically significant.

**Conclusions:**

The KM2H^2^ program showed a persistent effect up to 30 months post intervention in enhancing physical activity and reducing the risk of cardio-cerebrovascular events, particularly stroke. These findings demonstrate the persistent effect of the KM2H^2^ and suggest the need for a full-scale evaluation of the intervention program for practical use.

**Trial registration:**

ISRCTN Register ISRCTN12608966. Registered 03 March 2015. Retrospectively registered.

## Background

Preventing stroke and heart attack represents a very promising approach to reduce mortality from cardio- and cerebrovascular diseases [1]. Hypertension is one of the important modifiable risk factors, responsible for 45% of the deaths due to heart disease and 51% of the deaths due to stroke [2]. Along with rapid economic growth and the adaption of modern sedentary life styles, the prevalence of hypertension in China has increased 6.6 times in the past decade [3, 4]. The prevalence of hypertension increases to 56.6% for people 65 years of age and older [3, 4].

Antihypertensive medication has been used as a primary strategy to control blood pressure and prevent complications [5, 6]. Research has shown that antihypertensive drug therapy alone can reduce the risk of stroke by 30–47%, heart disease by 27–40%, and the overall cardiovascular disease mortality by 15% [7, 8]. The effect from antihypertension medication can be enhanced by physical activity. The role of physical activity in controlling blood pressure has long been recognized [9, 10]. Participating in regular moderate or higher levels of physical activity can also reduce other cardiovascular risks, such as weight gain, hypercholesterolemia, metabolic syndrome, and diabetes [11, 12]. However, few reported studies have examined the impact of physical activity on reducing the incidence of cardio- and cerebrovascular diseases among patients under antihypertensive medication.

A number of barriers may prevent senior patients with hypertension from engaging in physical activities at the community level [13, 14]. Hypertensive patients are often older in age and lower in self-confidence to engage in physical activity [15, 16]; these patients are also lack of adequate societal and familial supports [15, 16] to maintain recommended activity levels [17, 18]. In addition, senior hypertensive patients differ from each other dramatically. For example, some are retired while others are still working; some may live with spouse and children while others may live alone; some are better off while others live on social security funding.

Considering the barriers described above, a program “Keep Moving toward Healthy Heart & Healthy Brain” (KM2H^2^) was developed to promote senior hypotensive patients to engage in physical activities. The program is guided by the Transtheoretical Model (TTM) to encourage and monitor the intervention progress by stages from pre-contemplation, to contemplation, preparation, action, and maintenance [19]. Our adaption of TTM is based on the existing studies and other intervention programs conducted among Chinese populations [16, 19–21]. Considering the large between-patient differences, the KM2H^2^ has incorporated a one-on-one consulting strategy guided by the Model of Personalized Medicine to enhance tailored precision intervention [22, 23]. To achieve long-term effect, a group-based intervention strategy is used with guidance of the social capital theory [24]. With group-based sessions delivered in the community settings, the KM2H^2^ provides opportunities for the participants to develop network connections to enhance social and emotional supports for physical activity initiation and maintenance [24, 25].

The intervention modules of KM2H^2^ and the measurements of physical activity were established through a series of rigorous pilot tests [26]. In a previous evaluation study, we demonstrated that participants who received KM2H^2^ were more likely to engage in physical activity at 3-month and 6-month post intervention and less likely to experience stroke and heart attack at 6-month post intervention [27]. The purpose of this study is to evaluate if the observed effects of KM2H^2^ on encouraging physical activity and reducing the risk of cardio- and cerebrovascular events persist at 30-month post intervention.

## Methods

### Design of the trial

Design of the trial has been descried in detail elsewhere when reporting the effect of KM2H^2^ at 3- and 6-month post intervention [27]. Briefly, a two-arm cluster randomized controlled trial was used. When planning the trial, power analysis indicated the need for a sample of 440~480 patients to ensure 80% power to detect a moderate effect size with alpha at 0.05 level (2-sided). Key factors such as the number of clusters, cluster size and waves of data collection were considered in the power analysis [28]. Participants were selected from Wuhan, a provincial capital city located in central China.

**Fig. 1:**
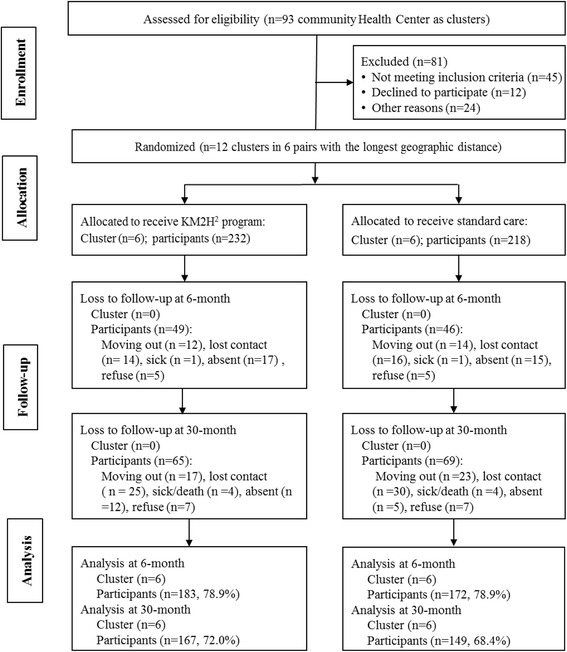
CONSORT flow chart of participants

Following the design, a total of 12 community health centers from the city of Wuhan were identified and grouped into 6 pairs with largest geographic distance and similar socioeconomic conditions. Approximate 50 participants per center were enrolled. The enrolled participants were randomized by center pairs to receive either KM2H^2^ plus standard care as the intervention group or standard care only as the control group. A total of 586 hypertensive patients were approached, 450 agreed to participate with 232 from 6 centers to receive the intervention and 218 from 6 centers to receive the control condition. Data collected at the baseline, 3-, 6- and 30-month post intervention were included in this analysis. More detailed information regarding the research design, follow-up assessments and attrition was presented in Fig. 1.

The intervention trial was approved by the corresponding Institutional Review Boards at Wuhan Center for Disease Prevention and Control (for conducting the trial, collecting the baseline and all follow-up data, and participating in statistical analysis and manuscript development), Hong Kong Polytechnic University (for initial funding support to initiate the project and for participation in the intervention program development) and the University of Florida (for statistical analysis of the collected data and the development of the manuscript for publication). All participants provided written informed consent before they were included in the trial.

### Participants, inclusion and exclusion criteria

Participants were recruited among patients who were enrolled in a Community-Based Hypertension Control Program (CBHCP) of Wuhan. The CBHCP was launched by Wuhan CDC in 2006 to provide antihypertensive drug therapy for all residents who were diagnosed with hypertension. In addition to the provision of relevant medication, standard care was provided to individual patients on a regular basis, including health education about blood pressure management, dietary, and physical activities, as well as follow-up checkups on blood pressure, side effect of medication, and any other related issues. The standard care services were provided at the community health centers.

Participants were recruited during January 20 and November 28, 2012. The inclusion criteria were: 1) 55 years of age or older, 2) diagnosed with hypertension and currently on antihypertensive medication, 3) be able to complete the planned intervention activities, 4) no history of stroke and heart attack, and 5) can safely complete the recommended moderate or higher levels of physical activities.

The following criteria were imposed to exclude participants who 1) were failed to the Physical Activity Readiness Questionnaire test [29], 2) suffered from obvious heart dysfunction, 3) had other physical, mental, and medical conditions that prevented them from completing the required research activities.

### Intervention program KM2H^2^

**Fig. 2:**
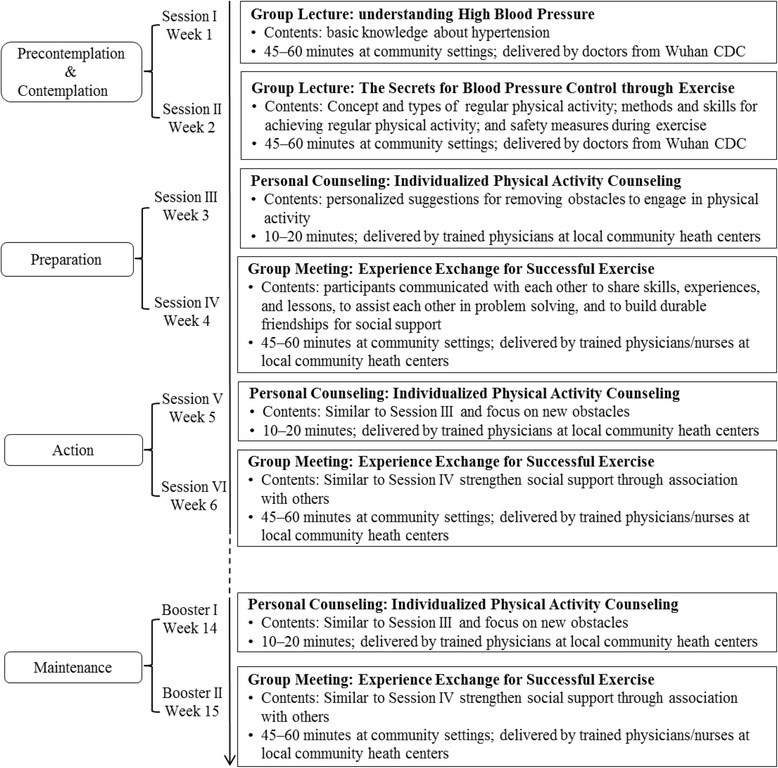
Six intervention sessions and two booster sessions in KM2H^2^ program

The intervention program KM2H^2^ was briefed in the Introduction and elsewhere [27]. The program is guided by a multi-theory conceptual framework, consisting of TTM [30], Model of Personalized Medicine [22, 23], and social capital theory [24]. The KM2H^2^ consists of six sessions, plus two booster sessions (Fig. 2). The six intervention sessions were delivered on a weekly basis during the first six weeks, including two group lectures (Session I and II), two personal counseling sessions (Session III and V), and two small group meetings (Session IV and VI). With these planned intervention sessions, participants were expected to move across the five TTM stages from Precontemplation (not ready), to Contemplation (getting ready), Preparation (ready), Action and finally Maintenance to engage in the enlisted physical activities.

Session I covers the basic knowledge of hypertension, risk and protective factors, and blood pressure self-management. Sessions II focuses on skills training, including the concept and types of regular physical activity, methods and skills for achieving regular physical activity, and safety measures for exercise. Participants were also asked to identify barriers that might prevent them from engaging in physical activities. The two sessions were delivered at community health centers by preventive medical doctors from Wuhan CDC. The two lecture sessions aim to assist participants in evaluating pros and cons of engaging in physical activity and promoting positive behavior expectations.

After the two lecture sessions, participants were fostered to make plans for physical activity. To assist individual participant in preparing and initiating regular physical activity, an  one-on-one telephone counseling was provided at week three, followed by a small group meeting session at week four. The telephone counseling was delivered by trained physicians to solve problems identified through the first two sessions that might prevent participants from engaging in physical activity. The small group meeting (8–10 patients per group) was initiated and supervised by the trained physicians/nurses at community health centers. For each group, a group leader was elected by all participants of the group to coordinate the required activities. Through group activities, participants in a group were expected to build durable and trustworthy network connection, share successful experience and lessons on engaging in physical activities, ensure safety, and overcome barriers for physical activity.

At week five, a second one-on-one telephone counseling (session V) was delivered to help remove new obstacles that might have prevented a participant from engaging in physical activity continuously. A second group meeting (session VI) was arranged in the week six to further strengthen their social network connection to support long-term intervention effects and maintain the established physical activities.

Since the purpose of the KM2H^2^ is to establish a new active lifestyle, two booster sessions were added for longer-term behavior maintenance, including a telephone one-on-one counseling session at week 14, followed by a group meeting session at week 15. The personal telephone counseling was used to solve additional problems confronted by individual participant since the last session in week six; the group session was designated to further the social connection for information exchanging, experience sharing, and mutual supporting.

To ensure the fidelity of the intervention, we selected the interventionists among senior staffs from Wuhan CDC who were experienced in implanting behavioral interventions, including lecturing, telephone consulting, and group-activity organization. We also limited the number of interventionists to minimum, with one person to deliver the lecture and organize group activities and one for telephone consulting. The interventionists received rigorous training and all interventional activities were conducted following the established protocol.

### Data collection, variables and their measurement

#### Data collection

To the time of this analysis, four waves of data were collected, including the baseline assessment prior to the KM2H^2^ delivery, and follow-up assessments at 3, 6 and 30 months post intervention. Survey data were collected using self-reported questionnaire through in-person interview. All data collectors, including graduate students, physicians, nurses and staffs were trained according to a standard data collection protocol. The survey was administrated separately by trained graduate students who were blinded from the intervention conditions. Clinical data, including family history, height and weight, and blood pressure were obtained from patients medical records. The medical records were generated through routine clinical practice by physicians and nurses who were also blinded from the intervention conditions. Participants were compensated with a free meal (equivalent to ~$3) upon completion of each wave of survey data collection.

#### Events of stroke and heart attack

Stroke and heart attack were the primary outcomes of this study. As indicated in the inclusion criteria early in this paper, only participants with no heart attack and/or stroke at baseline were included in the analysis. New heart attack/stroke events were identified and determined using a two-step strategy – patients’ self-report through in-person interview first, and then verified by medical records from the corresponding CBHCP community health centers. At all assessments, participants were asked two questions, one related to stroke and another on heart attack: “Since last interview, have you had stroke (heart attack) or diagnosed by your doctor that you had a stroke (heart attack)?” For patients who responded positively to a question, the trained data collectors confirmed the reported results against their medical record stored in the corresponding CBHCP community health centers. Only new events of stroke and heart attack during the follow-up period were included in analysis.

#### Physical activity

At each assessment, participants were asked to mark the physical activities they did in the past week using a standard list of physical activities, which was established based on the recommend definition of regular physical activity and our rigorous pilot tests [26, 31]. The list includes brisk walk, tai chi, jogging, bicycling, swimming, floor sweeping, house cleaning and any other activities by participants’ assessment that has the same intensity. Participants who reported participating in a physical activity were further asked to report the number of times they had engaged in the activity in the past week and the average duration per day using a 5-point rating scale (0: no exercise, 1: <15 min, 2: 15–29 min, 3: 30–59 min, and 4: 1 h or more). Qualified regular physical activity and duration were created using these reported data. Regular physical activity was defined as currently engaging in the designated physical activities for at least 30 min per day and for at least 3–5 times per week. For participants who qualified as engagement in regular physical activity, their duration of excise was measured using the 5-point index score with 0 = zero minutes and 4 = 1 h or more. This duration measure, although semi-quantitative, showed adequate validity and frequently used in published studies [26, 32, 33].

#### Blood pressure

Blood pressure (mmHg) was measured as part of the standard care of CBHCP by registered nurses. These nurses were blinded from the intervention status of individual participants. The measurement of blood pressure was completed following standard procedure recommended by the American Heart Association [34], and mean blood pressure of two separate measures at least 3 min apart was used in analysis. Both systolic and diastolic blood pressures were recorded.

#### Covariates

Covariates included age, sex (male and female), marital status (married/cohabit vs. divorced/separated), monthly income (RMB), regular cigarette smoking (yes/no), regularly alcohol consumption (yes/no), years of hypertension, adherence to antihypertensive medication, and family history of cardiovascular disease.

### Statistics methods

Chi-square test (for binary variables) and t-test (for continuous variables) were used to assess baseline comparability of the intervention group and the control group. Multivariate methods for longitudinal data, including mixed effects modeling (for continuous variables) and generalized linear mixed effects modeling (for binary variables) were used to assess the effects of the KM2H^2^ on enhancing physical activities and reducing risk of cardio- cerebrovascular events, controlling for the design effect, including cluster randomization and repeated measures, baseline difference between intervention and control, and covariates. These two multivariate modeling methods are robust to handle missing data due to follow-up attrition and capable of estimating adjusted outcome measures and their 95% confidence intervals. With a multivariate mixed effect modeling approach, the interaction between time and intervention conditions provides a rigorous measure of the efficacy of our intervention program KM2H^2^. Statistical analyses were completed using commercial software SAS version9.30. As usual, type I error was set at *p* < 0.05 (two-tailed) for statistical inference.

## Results

### Characteristics of the study participants

Of the total 450 participants, 355(78.9%) completed the assessment at 6 months follow-up, including 183 (78.9%) from the intervention group and 172 (78.9%) from the control group; 316 (70.2%) completed the assessment at 30 months post intervention with 167 (72.0%) from the intervention group and 149 (68.4%) from the control group. Comparative analysis indicated no significant differences in demographic and other factors between those who were lost to follow-up and those who remained in the trial with one exception - participants who were lost were older than those who remained in the study. Reasons for lost to follow-up were summarized in Fig. 1. Baseline characteristics of the study participants were reported in the previous study [27]. We presented these data again for the convenience of reviewing (Table 1).

**Table 1:** Baseline characteristics of participants in KM2H^2^ program

Characteristic	Total	Intervention	Control	*p* value
Sample size	450	232	218	
Age in years, *n* (%)				0.662
55–59 years	119 (26.4)	53 (22.8)	66 (30.3)	
60–64 years	136 (30.2)	76 (32.8)	60 (27.5)	
65–69 years	111 (24.7)	66 (28.4)	45 (20.6)	
70+ years	84 (18.7)	37 (16.0)	47 (21.6)	
Mean (SD)	64.2 (6.1)	64.3(5.6)	64.0 (6.5)	
Gender, *n* (%)				0.625
Male	189 (42.0)	100 (43.1)	89 (40.8)	
Female	261 (58.0)	132 (56.9)	129 (59.2)	
Monthly personal income RMB, *n* (%)				0.001
<1000 Yuan	94 (20.9)	42 (9.3)	52 (23.8)	
1000–2499 Yuan	234 (52.0)	111 (47.8)	123 (56.4)	
≥2500 Yuan	115 (25.6)	77 (33.2)	38 (17.4)	
Unknown	7 (0.9)	2 (0.9)	5 (2.3)	
Marital status, *n* (%)				0.002
Married/cohabit	398 (88.4)	216 (93.1)	182 (83.5)	
Divorced/separated	36 (8.0)	10 (4.3)	26 (11.9)	
Not reported	16 (3.6)	6 (2.6)	10 (4.6)	
Regular smoking, *n* (%)				0.004
Yes	85 (18.9)	32 (13.8)	53 (24.3)	
No	365 (81.1)	200 (86.2)	165 (75.7)	
Regular drinking, *n* (%)				0.952
Yes	80 (17.8)	41 (17.7)	39 (17.9)	
No	370 (82.2)	191 (82.3)	179 (82.1)	
Blood pressure (mmHg)				
Systolic, mean (SD)	141.2 (17.2)	140.7 (17.1)	141.7 (17.3)	0.565
Diastolic, mean (SD)	86.5 (10.8)	86.1(9.3)	87.0 (12.3)	0.399
Years of hypertension, *n* (%)				0.021
<5 years	143 (31.8)	61 (26.3)	82 (37.6)	
5–14 years	184 (40.9)	98 (42.2)	86 (39.5)	
≥15 years	123 (27.3)	73 (31.5)	50 (22.9)	
Mean (SD)	12.5 (10.7)	13.0 (10.5)	12.0 (10.9)	
Family history, *n* (%)				
Hypertension	264 (58.7)	141 (60.8)	123 (56.4)	0.349
Diabetes	48 (10.7)	25 (10.8)	23 (10.6)	0.938
Stroke	68 (15.1)	41 (17.7)	27 (12.4)	0.118
Heart diseases	52 (11.6)	32 (13.8)	20 (9.2)	0.126
Hyperlipidemia	34 (7.6)	16 (6.9)	18 (8.3)	0.585
Medication adherence, *n* (%)	314 (69.8)	161 (69. 4)	153 (70.2)	0.856

Note: *SD* standard error

### Intervention effect on stroke

**Fig. 3:**
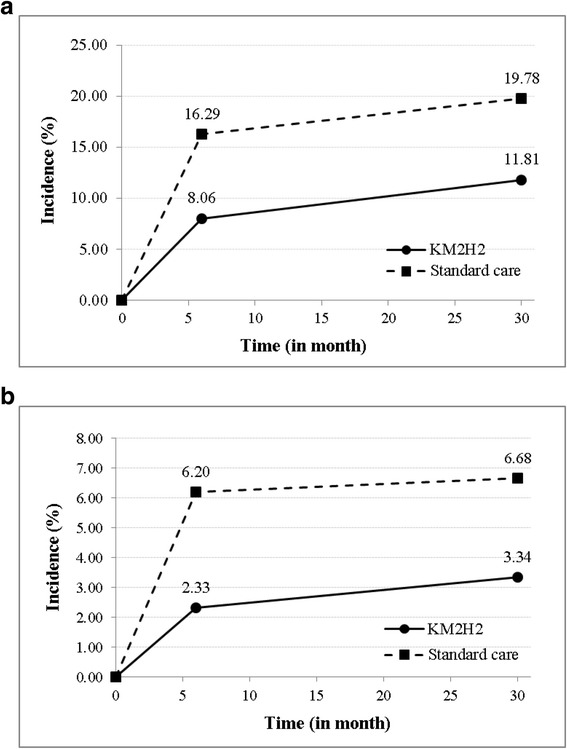
Differences in the adjusted incidence of stroke and heart attack between patients receiving KM2H^2^ and the standard care at 6- and 30-month post intervention. Note: **a**: the adjusted incidence of stroke; **b**: the adjusted incidence of heart attack. The adjusted incidence was estimated and compared using generalized linear mixed effects model controlling for effect of cluster randomization, repeated measures, baseline difference, and other covariates

Up to the 30-month post intervention, 19 (11.18%) new stroke cases among the 170 intervention participants were reported, including 2 new cases since 6-month post-intervention. Among the 157 participants in the control group, 43 (27.39%) new stroke cases were reported, including 5 new cases since 6-month post intervention. Results in Fig. 3a from the generalized linear mixed effects modeling analysis indicated that the adjusted 30-month incidence rate [95% CI] was 11.81% [5.90, 17.72] for the intervention group and 19.78% [14.07, 25.50] for the control group. The model coefficient [95% CI] for the time*intervention term of the generalized linear mixed effect modeling was −0.06 [−0.09, −0.02] (*p* = 0.03). The results were obtained after controlling for the complex design effect, baseline differences and the key covariates.

### Intervention effect on heart attack

Up to the 30-month post intervention, among the 169 intervention participants 9 (5.33%) new heart attack cases were reported, including 1 new case since the 6 months post intervention. Among the 154 participants in the control group, 16 (10.38%) new heart attack cases were reported during the same period with no new cases since the month 6. Results from the generalized linear mixed effects modeling analysis in Fig. 3b indicated that the adjusted 30-month incidence rate [95% CI] was 3.34% [1.91, 8.58] for the intervention group and 6.68% [1.64, 11.73] for the control group. The model coefficient [95% CI] for the time*intervention term of the generalized linear mixed effects modeling was −0.03 [−0.06, 0.01] (*p* = 0.16).

### Intervention effect on physical activity

Results from the upper panel of Table 2 indicate that at 30-month post intervention, 64.98% [54.81, 75.16] of the intervention patients maintained regular level of physical activity, while the adjusted proportion was 40.97% [30.78, 51.16] for control patients. The model coefficient [95% CI] for the time*intervention term of the mixed effects modeling was 0.24 [0.14, 0.34] (*p* < 0.01).

**Table 2:** Differences in regular physical activity between the KM2H^2^ and the standard care groups at baseline, 6-month and 30-month post intervention

Variables/Times	KM2H^2^		Control		*p* value
Regular physical activity	Crude	Adjusted	Crude	Adjusted	
	% (n/N)	% [95% CI]	% (n/N)	% [95% CI]	
Baseline	56.28 (130/231)	54.26 [44.84,63.68]	48.58 (103/212)	45.68 [36.38,54.98]	0.08
6-month	79.23 (145/183)	75.57 [65.60, 85.54]	42.69 (73/171)	42.12 [32.22,52.03]	<0.01
30-month	65.27 (109/167)	64.98 [54.81,75.16]	43.62 (65/149)	40.97 [30.78,51.16]	<0.01
Duration of regular physical activity	Crude	Adjusted	Crude	Adjusted	
	Mean (SD)	Mean [95% CI]	Mean (SD)	Mean [95% CI]	
Baseline	2.93 (1.20)	2.71 [2.43,2.99]	2.84 (1.12)	2.74 [2.46,3.02]	0.87
6-month	3.48 (0.99)	3.34 [3.05,3.62]	2.97 (1.35)	2.84 [2.54,3.12]	<0.01
30-month	3.00 (1.85)	2.83 [2.54,3.12]	2.64 (1.23)	2.46 [2.17,2.76]	0.04

Note: *SD* standard error, *CI* confidence interval. The adjusted proportion and mean were estimated and compared by using general linear mixed effects model (regular physical activity) and mixed effects model (duration of regular physical activity) controlling for effect of cluster randomization, repeated measures, baseline difference, and covariates

Results in the bottom panel of Table 2 indicated that the mean exercise duration index [95% CI] was 2.83 [2.54, 3.12] for the intervention group and 2.46 [2.17, 2.76] for the control group. The model coefficient [95% CI] for the time*intervention term of the mixed effects modeling was 0.36 [0.03, 0.69] (*p* = 0.04).

It is worth noting that the level of physical activity for patients in the control group was relatively high. This is because before participating in this trial, both the intervention and control patients received standard care as part of the CBHCP with physical activity being included.

### Intervention effect on blood pressure

Results from the Table 3 indicate that at 30-month post intervention, the average systolic blood pressure [95% CI] was 132.15 mmHg [127.90, 136.39] for intervention group and 135.17 mmHg [130.90, 139.43] for control group; the average diastolic blood pressure [95% CI] was 78.22 mmHg [75.21, 81.23] for intervention group and 79.82 mmHg [76.80, 82.83] for control group. The differences between the two groups for both systolic and diastolic blood pressure were not statistically significant at *p* < 0.05 level.

**Table 3:** Systolic and diastolic blood pressure in the KM2H^2^ and standard care groups at baseline, 6-month, and 30-month post intervention

Variables/Times	KM2H^2^		Control		*p* value
	Crude Mean (SD)	Adjusted Mean [95% CI]	Crude Mean (SD)	Adjusted Mean [95% CI]	
Systolic BP					
Baseline	140.72 (17.14)	140.70 [136.72,144.69]	141.67 (17.30)	140.56 [136.67,144.44]	0.94
6-month	136.15 (16.00)	134.82 [130,41,139.23]	138.38 (15.31)	137.26 [133.12,141.40]	0.31
30-month	132.71 (13.69)	132.15[127.90,136.39]	136.00 (16.09)	135.17[130.90,139.43]	0.21
Diastolic BP					
Baseline	86.11 (9.32)	84.27 [81.40,87.15]	86.99 (12.26)	85.67 [82.86,88.50]	0.39
6-month	81.19 (9.17)	79.03 [75.92,82.13]	84.50 (10.03)	83.40 [80.44,86.36]	0.11
30-month	80.28 (8.69)	78.22 [75.21,81.23]	81.82 (8.56)	79.82 [76.80,82.83]	0.37

Note: *SD* standard error, *CI* confidence interval, *BP* blood pressure. The adjusted mean were estimated and compared by using mixed effects model controlling for the effect of cluster randomization, repeated measures, baseline difference, and covariates

## Discussion

The intervention program KM2H^2^ is a theory-guided and community-based behavioral intervention for senior hypertensive patients. It is developed with guidance of an integrative theoretical model including the TTM, the Model of Personalized Medicine and the social capital theory. This program was implemented to enhance physical activity among senior patients on antihypertension medication and improve their control of blood pressure, further reducing the risk for stroke and heart attack. Findings of this study and previous evaluation study [27] indicate the intervention effects of the KM2H^2^ largely persisted up to 30 months post intervention. There is a lack of physical activity intervention program for the senior hypertensive patients in China. The KM2H^2^ is the first study that shows significant effects up to 30 months post-intervention in improving physical activity and reducing the risk of stroke and heart attack among this vulnerable population.

Findings of study indicate that appropriate level and frequency of physical activity can significantly reduce the risk of stroke and heart attack, the effect can be maintained for two and half years. The effects of the KM2H^2^ in reducing the risk of stroke and heart attack is in consistent with that of reported studies by others showing negative associations between physical activities and the morbidity and mortality of various cardiovascular diseases [35–37]. The cost for delivering the intervention is rather smaller relative to the medication for the antihypertension treatment; but the effect is substantial. A full-scale phase III trial is necessary to assess the effectiveness of the KM2H^2^ program for a wider use.

Receiving the KM2H^2^ was associated with 3.02 mmHg and 1.60 mmHg reductions in the systolic and diastolic blood pressure, respectively while the reductions in blood pressure were not statistically significant. This appears to be inconsistent with other physical activity intervention program that showed a significant effect of physical activity on reducing elevated blood pressures [38–40]. A possible reason is that the majority of published studies were conducted among adolescents and adults while the participants of our study were senior hypertensive patients. Hypertensive patients at older age have undergone degenerative changes in their cardiac/cerebra vascular systems [41]. It is much harder to reverse these age-related pathogenic changes through physical activity alone [42].

In supporting the reductions in the risk of stroke and heart attack, the KM2H^2^ showed persistent effect in encouraging and maintaining physical activities among senior hypertensive patients who are under antihypertensive medication. The significant and sustained program effect has been observed at all follow-up assessments. The proportion of patients engaging in physical activity at baseline was comparable between intervention and control group (54.26% vs. 45.68%). By 6 months post-intervention, 21.3% of participants in the intervention group initiated regular physical activity (Action stage) while none in the control group did so. At 30 months post intervention, 64.98% of the intervention participants engaged in and maintained regular physical activity compared to 40.97% for the control participants. Although the guiding role of the TTM has also been challenged by the lack of observed intervention effect for physical activity intervention [30], findings from our research indicate that inclusion of social capital-based intervention and personalized consulting provides a remedy.

There are several strengths with our study. First, our intervention program KM2H^2^ is established through a series of rigorous pilot tests. By conducting these pilot studies, we identified appropriate barriers (e.g. lack of knowledge, psychosocial support, and self-confidence) and facilitators (e.g. some interests and willingness to participate if offered by health professionals and not for commercial purpose) for physical activity [26]. These data are essential for targeted interventions. Second, our intervention program emphasizes both the *precision medicine-based* personal intervention and the *social capital theory-based* group intervention [22–24]. The personalized intervention part enables us to solve problems for specific individuals, while the group-based intervention encourages social cohesion and social support to achieve long-lasting program effect. It shows a promising result in encouragement for engaging in physical activity at community level. Further, there are few physical activity promotion programs in China targeting for older persons who are affected most by CVDs, particularly for the hypertensive senior adults. Lastly, the intervention effect was evaluated using the rigorous mixed effects modeling methods, controlling for the influence of cluster randomization, repeated measurement, as well as potential baseline differences and key covariates that are inherited from the cluster randomization design.

There are limitations to this study. No objective measures were used to assess physical activities. Although the methods we used are pilot-tested and appropriate for community-based trials [43, 44], no criterion validity was established. More objective measures of physical activity such as wearable pedometers should be used in future study. In addition, the protocol used to determine stroke and heart attack could be further improved. Although patient-report plus medical record verification has often been used in community-based research [45], miss report is likely. Like in many trials, not all participants were assessed at 30 months post intervention and the attrition rates may differ for the intervention and control groups. Despite the use of advanced mixed effects modeling method for data analysis, bias cannot be fully ruled out. Lastly, the participants of this trial are recruited from one city in China, caution is necessary when interpreting our findings to other population.

## Conclusions

Despite these limitations, this study demonstrates the efficacy of the KM2H^2^ program in successfully encouraging physical activity and reducing the risk of stroke and heart attack among senior hypertensive patients up to 30 months post intervention. These findings underscore the urgency for a full-scale trial to establish the utility of KM2H^2^. In the full scale trial, a number of issues must be considered, such as objective measuring for physical activity and rigorous protocol to determine stroke and heart attack. In addition, training for community health workers for program delivery should also be emphasized.
